# Notes and News

**Published:** 1887-07-09

**Authors:** 


### NOTES AND NEWS.

COLLECTED AND COMMUNICATED.

AT Burslem, in Staffordshire, the Haywood Hospital was formally opened on Monday for the reception of the sick.

MISS LIVERMORE has recently presented a valuable water-bed to the Chelmsford and West Essex Infirmary. Many a patient will bless Miss Livermore in future years. Mrs. Ridley, of The Glens, has sent a package of New Testaments as a Jubilee offering.

THE rector of Christ Church, Blackfriars Road, has 800 poor children in his parish, all of whom he would like to send into the country if he had the means. Those who have money to spare cannot do better than send something to the rector for this object.

AT King's Norton parish church a flower-service has been held, and an address given by the vicar, Rev. W. Preedy. A collection, amounting to £1 7*s.*, was made, which, together with the flowers, was transmitted to the Children's Hospital, Birmingham.

COLLECTION-BOXES are placed by the managers of the Richmond Hospital at various points of vantage in the district, and are opened half-yearly in June and January. The total amount collected in all the boxes during the last six months was £120 4*s.*—a not inconsiderable addition to the resources of the hospital.

THE hospital of the little town of Retford, on the Great Northern Railway, has just issued its annual report, which shows a most satisfactory amount of work well done, and a prosperous condition of the funds.

MESSRS. COOPER COOPER AND CO. have forwarded to Mrs. Morris a chest of tea as a donation to the bazaar, in aid of the South End Victoria Hospital; and Messrs. Huntley and Palmer have sent a case of fancy biscuits. These are good examples, which might be imitated on an extensive scale.

FROM their Jubilee Tea Meeting the patients at the Edinburgh Royal Infirmary sent a telegram to the Queen on Jubilee day. Her Majesty, also by telegram, returned the following gracious reply:—"The Queen thanks the patients of the Royal Infirmary, Edinburgh, for their kind and loyal congratulations."

SIR W. T. LEWIS on Jubilee Day laid the foundation stone of a new hospital at Merthyr. The Marquis of Bute gives a further sum of £1,000 in addition to what he has already subscribed. 35,000 pence have been handed over to the fund by the miners of the neighbourhood.

ON Tuesday, in splendid weather, the foundation stone of the additional wards to the Gravesend Hospital was laid by Mrs. J. Russell. the munificent donor of the new buildings. There was a large and distinguished company present.

THE vicar of St. Philip's, Bethnal Green, is in urgent need of assistance for his school children, many of whom are the poorest of the poor, and whom he wishes to send into the country for a day or more. A few shillings will send a child for a whole week. Who will help?

MR. W. T. SMEDLEY, Honorary Secretary to the Birmingham Hospital Saturday Fund, is about to receive a handsome testimonial in recognition of his long and valuable services on behalf of the Fund. The testimonial is to take the form of an illuminated address, and a purse of gold.

THE trustees of Westminster Hospital have just received a Jubilee presentation in the shape of a half-length portrait of the Queen, painted in water-colours by Miss L. Campbell Clark, from a print of her Majesty about the year 1840. The portrait, which is admirably executed, is contained in a Florentine gold frame, and is specially intended for the children's ward at Westminster Hospital. It has been gratefully accepted by the trustees.

THE following vacancies are announced this week, the amount of the salary being placed in each case after the name of the institution :—

**Resident Medical Officers.**

Birmingham Queen's Hospital. Casualty Surgeon. Qualified.—£50.
City of London Hospital for Diseases of the Chest. Clinical Assistant. Qualified.
Royal Hospital for Children and Women, S.E. Physician. Qualified.
Southport Infirmary. House Surgeon. Doubly-qualified. —£100.
Stafford Infirmary. Assistant House Surgeon.—No salary.

**Non-resident Medical Officers.**

Bristol Royal Infirmary. Obstetric Physician.
Kilburn Dispensary, 13, Kilburn Park Road. One vacancy.
Reeth Union. Medical Officer. Duly qualified.
Sedburgh Union. Medical Officer. Doubly-qualified.—£20
Truro Union. Medical Officer. Qualified.—£20.

**Matron.**

Royal Sea Bathing Infirmary, Margate.—£85.

**Housekeeper.**

York Retreat.—£60.

**Trained Nurses.**

Establishment for Invalid Gentlewomen. One.—£25.
Greenwich Seamen's Hospital. One, night.—£20.
Maidstone, Monckton Home. Several.
Maidstone General Hospital. Lady Nurse.—£25.

MR. PERCY TEW appeals to the Wakefield public for help to pay off a debt on the Clayton Hospital of that town. This is the only medical charity in the town of any importance. A few of the principal inhabitants have offered liberal subscriptions, but the list is very short, and the names of many prosperous residents are conspicuous by their absence, notably those of some of the former mayors of the borough.

THE Duke of Cambridge has paid a visit to the Royal Hospital, Chelsea, to unveil a bust of the Queen, in commemoration of the Jubilee. The idea of thus giving expression to the loyalty of the veteran inmates originated with the Governor, Sir Patrick Grant. The Duke, after a few stirring words from Sir Patrick, made a short speech, and unveiled the bust. Three ringing cheers were give for the Queen and three for the Duke, when the ceremony was over. The bust, by Mr. Boehm, is an excellent likeness of her Majesty.

THE *John o' Groat Journal* says : "On the annual holiday a number of ladies and gentlemen paid a visit to the Edinburgh Hospital, Thurso, under the charge of Mr. and Mrs. Chisholm. Everything was found to be in 'apple-pie order,' and the only regret felt was that its excellent advantages were not embraced by those to whom it would be an inestimable boon." Are we to understand that the "Edinburgh Hospital at Thurso" has a superintendent and matron, but no patients ?

THE residents of Greenwich, Deptford, Lewisham, Blackheath, Kidbrook, and Charlton are being solicited to aid in the establishment of a free ophthalmic hospital for the district. This is a step in the right direction. To increase the number of special hospitals in the metropolis is worse than carrying coals to Newcastle, but to plant them here and there in the midst of dense populations where they are really needed, is what all charitable and practical persons will thoroughly approve.

AT a meeting of the Council of The Hospitals Associations, held on Wednesday last, it was decided to issue the circular letter drawn up by the Sectional Committee on Nursing and Domestic Management to the lady superintendents or matrons of all the principal hospitals and infirmaries in the Kingdom, asking a series of questions on nursing and domestic arrangements. It is hoped that each lady communicated with will do her part to assist the Council by answering the questions as fully as possible. The result only, but no names, will be published.

THE Secretary of The Hospitals Association (Mr. Howard J. Collins) will be out of town until the 26th inst., and members and others are therefore requested to excuse the delay, should any occur, in replying to their communications. The office will be open for inquiries, etc., as usual.

THE female scarlet-fever wards of the London Fever Hospital, Islington, have just received a notable and very valuable addition in the shape of some decorative wall-paintings. These paintings consist of groups of flowers, painted on the varnished surface of the walls, and have been executed by Miss Emily Wall whilst a patient in the hospital. As works of art they are of a very high order of merit. Were it not for the danger of infection, it would be well worth the time to pay a visit to the hospital solely to see Miss Wall's flowers. The brightness and beauty they add to the ward it is hardly possible to overrate.

SIR JOHN LUBBOCK, M.P., F.R.S., distributed the prizes to the students of the Charing Cross Hospital Medical School. The secretary reports the School to be in a more flourishing condition than at any previous time. Speaking of the progress of medical science, Sir John said that many a poor sufferer was often restored to perfect health, who but a very short time ago would have been regarded as a hopeless case. The best physicians of the day were not more remarkable for their medical skill than for their charm of manner, their human kindness, and their warm sympathy with suffering. One man would do more without a single dose of medicine than another with the whole materia medica at his disposal.

MR. M. CURSHAM CORNER sends the folllowing suggestion to an evening contemporary *àpropos* of the threatened deadlock in hospital resources :

1. Persons only to receive the benefits of the hospitals who are deserving.
2. Cases of accident and emergencies only to be received without letters.
3. Donors of letters to use increased care before giving them to applicants.
4. Applicants to confine their attendance to hospitals in districts where they live.
5. Each hospital to have an area mapped out wherefrom patients can be received.
6. Governors not wishing to use letters, to give them to a charitable society.
7. All hospitals to publish a detailed balance sheet, and to be enjoined to practise greater economy.
8. A conference of managers to consider these various points.

On Saturday, 18th ult., a "Jubilee Grand Carnival State Procession," in aid of the Hospital Saturday Fund, was carried out in East London. The procession, which was about a mile and a half in length, formed in Victoria Park, marching thence, with bands and banners flying, to the People's Palace in the Mile-end Road. Numerous police bands and Temperance Societies assisted to swell the procession.

A good deal of disappointment has been felt in Sunderland at the inability of the Prince of Wales to lay the foundation stone of the Hartley Memorial wing to the Sunderland Infirmary. His Royal Highness states, in reply to the invitation sent him, that although he is anxious to visit this important town, his time is at present so much occupied that it will be impossible for him to lay the stone of the new Infirmary wing.

Thanks to the kindly forethought of the Dean of Westminster, the hospitals have come in for a timely bit of assistance. The "Repeated Jubilee Service" at the Abbey, which was due to a suggestion made by Dean Bradley to his clergy and the canons of Westminster, has realised the handsome sum of £2,500. The whole of this amount goes to the London hospitals;—one-third through the Hospital Sunday Fund, one-third to the Hospital Saturday Fund and Western Dispensary, and the remaining third direct to Westminster Hospital.

On Sunday week a "demonstration" of amalgamated Temperance Societies paraded the principal streets of Holloway, Highbury, and Tufnell Park, and afterwards proceeded to a piece of waste ground in Wedmore Street, Upper Holloway, where addresses were delivered. Here, and also along the line of route, collections were made in aid of the North London Nursing Association, of which the Archbishop of Canterbury, Sir Edmund Currie, and other prominent persons are patrons.

The Manchester Royal Infirmary is mourning the loss of Mr. Alderman Curtis, Mayor of the city, who for the past ten years has been an active member of the Board, and rendered invaluable service to the institution at a critical period in its history. The Board have conveyed to Mrs. Curtis and the family the assurance of their sincere condolence and deep sympathy.

At Montrose, the Rev. Mr. Cameron, at a meeting of the Infirmary Board, said, referring to Doctors Lawrence and Key, the Honorary Medical Officers,—"Neither of our present medical men are distinguished surgeons." Some of the inhabitants of Montrose seem to be decidedly indignant at the slight cast upon those gentlemen, who give their services gratuitously to the infirmary, and very properly so. One pertinently suggests that possibly the doctors of the Montrose Infirmary are not the only undistinguished professional men who live in Montrose. The Rev. Mr. Cameron himself is not particularly well known at a distance from the ancient borough in which he lives.

The directors of the Lewes Infirmary, in anticipation of receiving some £600 or £700 from the Jubilee Fund, are contemplating important structural improvements in their hospital building.

The North Eastern Hospital for Children, in the Hackney Road, which was established just ten years ago to afford medical and surgical relief to the sick children of the poor of this populous neighbourhood, is, like most other medical charities, hard pressed for funds, though its work is greatly increasing. Last year there were 14,083 new cases dealt with in the out-patients' department, as against 13,801 in 1885, while the number of in-patients (618), although the hospital was closed for one month for cleaning purposes, was larger than in any previous year.

Annie Thein sued the magistrates and town-councillors of Aberdeen, as proprietors and managers of the City Epidemic Hospital, for £150 damages in consequence of alleged improper treatment in the hospital. The town council pleaded that the action was excluded in respect of the provisions of the Public Health Act of 1867, not having been raised within two months of its cause. Lord Fraser upheld this plea, and dismissed the action.

It is stated that her Majesty will very probably confer some honour on Dr. Morell Mackenzie, in recognition of his successful treatment of the Crown Prince of Germany. In medical circles in Germany a good deal of acrimonious feeling has been manifested at the preference shown by the Crown Prince and Princess for an English doctor. Dr. Morell Mackenzie, who is recognised as the first living authority on diseases of the throat, received much of his training at the London Hospital, where he is still one of the physicians. Several allusions were made at the Hospital Sunday Fund Meetings to Dr. Morell Mackenzie's treatment of the Crown Prince, one speaker stating that he considered that at the present moment there was no one more indebted to our London hospitals than the Crown Prince himself.

Dr. Rentoul, of Liverpool, continues to assail the ears of the public with his views about provident dispensaries and the excessive liberality of hospitals. Whether Dr. Rentoul will have the courage and the steadfastness to persevere until hospital managers can be brought to co-operate for the bringing about of a better state of things, we do not know. One thing is certain, that the present want of union produces a most melancholy paralysis of the whole hospital system. What is the cause of such want of union? Undoubtedly selfishness is at the bottom of it: a selfishness which is as foolish and reprehensible in itself as it is injurious to hospitals and everybody connected with them. The subscribers to medical charities should insist upon it that those institutions which they favour shall join heartily with all other similar institutions with a view to the inception and universal adoption of some scheme whereby those who are needy shall be promptly helped, and those who are not needy shall be as promptly shown how to help themselves.

